# *MDM2* 285G>C and 344T>A gene variants and their association with hepatocellular carcinoma: a Moroccan case–control study

**DOI:** 10.1186/1750-9378-9-11

**Published:** 2014-04-07

**Authors:** Khadija Rebbani, Sayeh Ezzikouri, Agnès Marchio, Mostafa Kandil, Pascal Pineau, Soumaya Benjelloun

**Affiliations:** 1 Unité de Virologie, Laboratoire des Hépatites Virales, Institut Pasteur du Maroc 1, Place Louis Pasteur, 20360 Casablanca, Morocco; 2 Equipe d’Anthropogénétique et biotechnologies, Faculté des Sciences, Université Chouaïb Doukkali, El Jadida, Morocco; 3 Unité « Organisation nucléaire et oncogenèse », INSERM U993, Institut Pasteur, Paris, France

**Keywords:** Hepatocellular carcinoma, *MDM2*, Susceptibility, Polymorphism

## Abstract

**Background:**

*MDM2* gene polymorphisms 285G/C and 344 T/A are two single nucleotide polymorphisms (SNPs) recently identified as important variants that could influence the expression of *MDM2* gene through the modulation of transcription factors binding on the SNP309T/G. The 285C variant seems to present a geographically distinct distribution in humans and to be associated with a low cancer risk. In the present report, we studied the distribution of the three SNPs in a population with low liver cancer incidence.

**Methods:**

A group of 119 patients with hepatocellular carcinoma (HCC, 63.45 ± 12.59 year, 26–80) and another of 103 non-HCC controls (56 ± 10.82 year, 22–79) were enrolled to investigate association between *MDM2* polymorphisms and susceptibility to develop HCC. The three studied SNPs (285G/C, 309 T/G and 344 T/A) were genotyped using polymerase chain reaction and sequencing techniques.

**Results:**

Genotypes and alleles distributions of the three studied polymorphisms of *MDM2* were not significantly different between cases and controls. An increased risk of HCC development was found in case of 309G allele presence albeit without reaching the significance (29.8% vs 22.3%, OR = 1.48, 95% CI, 0.96-2.27, *p* = 0.073). In addition, neither 285C nor 344A *MDM2* variants were significantly associated with an increased risk of HCC (*p =* 0.688 and *p* = 1 respectively). Remarkably, we found that the supposedly Caucasian-specific 285C variant was present in 1% of the Moroccan population.

**Conclusions:**

This is the first study of the *MDM2* SNP285G/C and SNP344T/A polymorphisms in association with HCC development. In contrast with previous studies, showing that females carrying SNP285C variant have a significantly reduced risk of developing breast, ovarian and endometrial cancer, no significant modulation of HCC risk was found in a North-African population.

## Background

Hepatocellular carcinoma (HCC), is the most frequent primary liver tumor, with a high incidence in Africa and Asia, which makes it the fifth human malignancy and the third most common cause of cancer-related mortality [1,2]. It is well established that HCC results from a complex tumorigenic process that occurs on a background of genetic and epigenetic alterations where environment (hepatitis viruses, aflatoxins) and lifestyle (alcohol or tobacco intakes) represent specific risk factors [3-5]. Morocco is a country of intermediate endemicity for chronic infections with hepatitis B (HBV) and C (HCV) viruses [6-8], where a consistently low level of food contamination by aflatoxins B1 [9,10]. In addition, the level of alcohol consumption is known to be historically low in Moroccans [11]. Despite this medium prevalence of chronic hepatitis, still the most important risk factor of HCC worldwide, the incidence of liver cancer in Morocco is known to be one of the lowest in the world (1.2 case/10^5^ habitants, ASR) [12].

The low impact of environmental and lifestyle risk factors on HCC incidence in Morocco attenuates their role as confounding agents and represents, thus, a favorable situation to detect individual susceptibility. Actually, individual susceptibility to HCC has been already studied in various populations and germinal variants of several genes were earmarked as associated with susceptibility [13-15]. Beside the *TP53* gene and its Arg72Pro polymorphism, *MDM2* gene, a negative regulator of the former with the SNP 309 T > G in the promoter region, is associated with hepatocellular carcinoma [16]. This SNP was described for the first time by Bond *et al.* (2004), to be associated with accelerated tumor formation, and then studied in a multitude of cancers [17,18]. In HCC, the 309 T > G SNP was profiled in different ethnic groups. To sort out some inconsistencies, an interesting meta-analysis was conducted in 2012 [19]. Seven different studies, including one survey on the Moroccan population, were evaluated [16]. This meta-analysis confirms the predisposing effect of *MDM2* 309G variant to HCC [19]. Another polymorphism on *MDM2*, 344 T > A SNP was identified in 2004 as accelerating tumor formation [17]. Recently, a G > C SNP located 24 base pair upstream the 309G > T SNP was described concomitantly by Knappskog *et al*. and Paulin *et al.* This 285C variant was found to antagonize the binding effect of SNP309T on Sp1 transcription factor [20,21].

Thus, the present report aims to describe the profiling of the three *MDM2* SNPs together for the first time in HCC patients and non-HCC controls recruited in Morocco.

## Methods

After giving written informed consent for genetic testing, every participant was interviewed. The study protocol was evaluated and approved by the ethics Committee of the Faculty of Medicine of Casablanca and the study was conducted in accordance with the ethical guidelines of the 1975 Declaration of Helsinki as reflected in a priori approval by the institution's human research committee. A total of 119 HCC patients and 103 controls were analyzed for the *MDM2* SNPs 285 (rs117039649), 309 (rs2279744) and 344 (rs1196333). The controls did not have a previous history of cancer of any type. Blood was collected in EDTA tubes; sera were prepared for ELISA tests and leucocytes for DNA extraction. Serological markers for hepatitis viruses were tested with commercially available kits (Axsym, Abbott Diagnostics, Germany) for HBsAg, anti-HBc and anti-HCV for HCC patients and non-HCC controls.

Genomic DNA was isolated from whole blood leucocytes. After washing in Tris/EDTA (TE) buffer, samples were submitted to digestion in SDS/proteinase K buffer at 37°C for 16 h maximum, followed by phenol/chloroform extraction. DNA was then ethanol-precipitated and re-suspended in TE buffer, as described previously [16].

*MDM2* polymorphisms were genotyped using an amplification followed by sequence analysis (PCR-Sequencing). Briefly, a step-down amplification was performed at annealing temperature of 55°C. The polymerase chain reaction was performed with a set of primers forward 5’-CCCGGACGATATTGAACA-3’ and reverse 5’-AGAAGCCCAGACGGAAAC-3’. The fragment was amplified in a 25 ml reaction volume using 0.2 mM primers, 200 mM dNTP and 1 U Taq polymerase. PCR products were loaded on a 2% agarose gel stained with ethidium bromide and a 226-bp strip was revealed. Positive PCR products were purified using the Exonuclease I/Shrimp Alkaline Phosphatase (GE Healthcare) and sequenced using BigDye Terminator version 3.1 kits and an ABI PRISM 3130 DNA automated sequencer (Applied Biosystems, Foster City, CA, USA). Nucleotide sequences were analyzed using SeqScape®v2.5 software (Applied Biosystems, Foster City, CA, USA).

Using a Chi-square test, Departures from Hardy–Weinberg equilibrium were determined in all groups for each SNP genotype distribution. Mann–Whitney test was conducted to compare medians. The significance of associations between genotype/alleles variants and susceptibility to HCC were assessed using Chi-square and Fisher’s tests with an estimation of the risk by computing ORs and their 95% CIs. A *p-value* <0.05 was considered statistically significant. All tests were two-sided. Haplotype frequencies and pairwise differences between groups’ analyses were performed using Arlequin 3.0 software, on the base of permutation tests using the EM algorithm [22].

## Results

### Demographic features of subjects

In the present report, we studied three SNPs on *MDM2* gene, in 119 HCC patients (63.45 ± 12.59 year, 26–80) and 103 controls (56 ± 10.82 year, 22–79). Table 1 summarizes general characteristics of the studied groups. Over 63% of the patients were positive for anti-HCV antibodies when HBsAg was present in 10.1% of patients. Another subset of patients (33.6%) was negative for HBsAg but presented a history of HBV infection, and 3.4% of cases were coinfected with HBV/HCV. About 6% of cases had type 2 diabetes mellitus. The mean tumor diameter was 3.5 ± 2.6 cm. The group of controls includes 103 volunteers without any history of cancer.

**Table 1:** General and clinical data of HCC patients and controls groups

	**HCC patients (%) n = 119**	**Controls (%) n = 103**
**Age (Mean ± SD)**	63.45 ± 12.59	56 ± 10.82
**Sex ratio (M:F)**	1.53 (72/47)	1.29 (58/45)
**Ongoing co-infection HBV + HCV**	4 (3.4)	0
**Chronic hepatitis B (HBsAg+)**	12 (10.1)	0
**Chronic hepatitis C (anti-HCV+)**	76 (63.9)	0
**Resolved Hepatitis B (HBsAg-/anti-HBc+)**	40 (33.6)	30 (29.1)
**No history of hepatitis B or C**	15 (12.6)	73 (70.9)
**Type 2 Diabetes**	7 (5.9)	1 (0.9)

### Genotypes and alleles distribution

**Figure 1:**
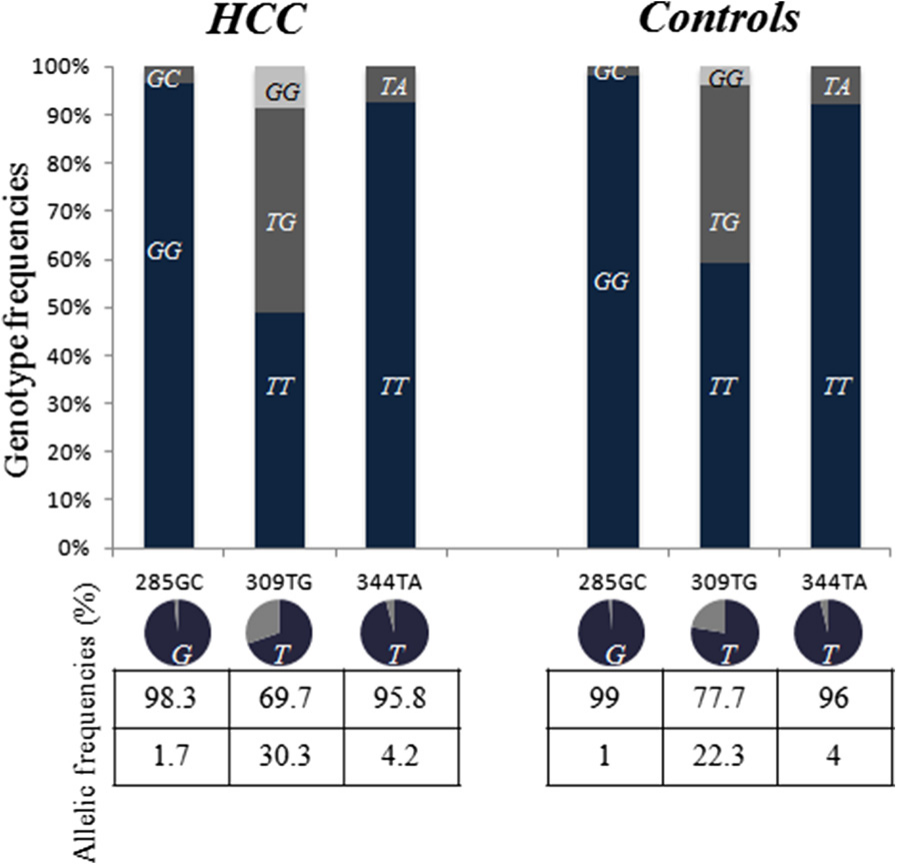
Genotype and allelic distribution of 285G/C, 309 T/G and 344A/T in Moroccans with and without HCC.

Allelic distributions in both patients and controls were in Hardy-Weinberg equilibrium (*p* > 0.05). Genotypes and alleles frequencies are presented in Figure 1. The first SNP (rs117039649) is a G/C substitution in position 285 of the MDM2 promoter region. The dominant genotypic form was GG with 96.6% of cases and 98% of controls, the remaining 3.4% of cases and 2% of controls were GC heterozygotes (OR = 1.757, 95% CI, 0.315-9.793, *p =* 0.688). Thus, out of the 222 profiled individuals, none present the genotype CC at rs117039649. For the rs2279744 (309 T/G), an increased risk of HCC was found in case of G allele presence. This risk did not reach, however, the level of significance (29.8% vs 22.3%, OR = 1.48, 95% CI, 0.96-2.27, *p* = 0.073). The same was true for patients carrying G/G genotype (OR = 2.63, 95% CI, 0.78-8.85, ns). The third SNP was rs1196333, an A/T substitution on the position 344. The major genotype was TT, whereas the TA genotype was present in 7.6% of cases and 7.8% of controls (OR = 1.118, 95% CI, 0.020-62.734, *p* = 1).

### Linkage disequilibrium and haplotype analysis

**Figure 2:**
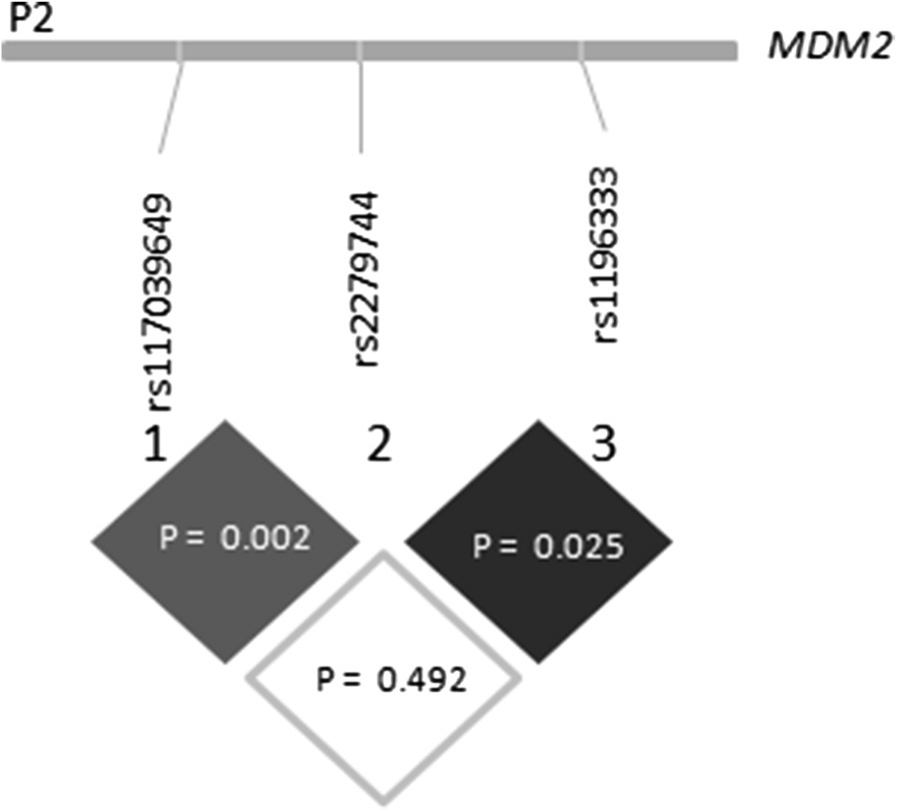
Pairwise linkage analysis of studied polymorphisms based on permutation tests using EM algorithm.

We calculated frequencies of genotypes combinations in both patients and controls for the three SNPs of *MDM2* in the order of SNPs on the chromosome 12q15 (rs117039649, rs2279744 and rs1196333). Only 7 and 6 out of 27 possible combinations were present in patients and in controls groups, respectively. In HCC patients, we observed 4 (3.4%) patients with 285GC genotype, all of them carrying at least a G at SNP309 and 344TT. On the opposite, the nine (7.6%) 344TA patients were 285GG and carrying at least a T at SNP309. Proportions were approximately the same in controls group and no significant haplotype association with HCC was found (*p* > 0.05, Table 2). The three SNPs are located within promoter P2 of *MDM2* gene, delimiting a region of 59 bp. A linkage analysis for these SNPs in 222 Moroccan individuals (HCC patients and controls) was performed (see Figure 2). Given the LD analysis, 285C is linked to 309G (*p* = 0.002), whereas the 344A is linked to 309 T (*p =* 0.025).

**Table 2:** Distribution of Genotype combinations of the SNPs 285G/C, 309 T/G and 344 T/A among HCC patients and controls

**Genotype combinations**	**HCC(%) n = 119**	**Controls (%) n = 103**
**GC/GG/TT**	1 (0.9)	0 (0.0)
**GC/TG/TT**	3 (2.5)	2 (1.9)
**GG/TG/TA**	2 (1.7)	2 (1.9)
**GG/GG/TT**	9 (7.6)	4 (3.9)
**GG/TG/TT**	46 (38.6)	34 (33)
**GG/TT/TA**	7 (5.9)	6 (5.8)
**GG/TT/TT**	51 (42.8)	55 (53.45)

Only four out of the eight possible haplotypes were observed in patients and controls. Rare haplotypes were present in the Moroccan population: a CGT haplotype (1%) and a GTA haplotype (3.9%). Table 3 shows haplotypes distribution in both patients and controls groups. Importantly, no haplotypic distribution difference was noticed between patients and control individuals.

**Table 3:** Haplotype frequencies for MDM2 gene SNPs 285G/C, 309 T/G and 344 T/A among Moroccans with and without HCC

**Haplotype frequencies (%)**	**HCC n = 119**	**Controls n = 103**	***p-values***
**GTT**	66.8	73.7	0.244
**GGT**	27.7	21.4	0.281
**GTA**	3.8	3.9	1
**CGT**	1.7	1	1

## Discussion

Research on HCC came through various eras of genetic and molecular advances but our understanding of the mechanistic process driving liver carcinogenesis remains incomplete due to the tremendous heterogeneity of this disease. Many studies focused on the p53 pathway considered as the major target undergoing aberrations in liver carcinogenesis. One of the most important regulators of the pathway is MDM2 (Mouse double minute 2 homolog), an E3 ubiquitin ligase that targets the p53 protein for proteasomal degradation. Several studies have shown previously that polymorphic variants on *MDM2* gene inversely correlate with *TP53* mutation status [23]. Recently, new SNPs on *MDM2* were discovered and shown to be associated with different cancer types [24,25].

In the present report, we investigate, for the first time, the potential role of SNPs 285G/C (rs117039649) and 344 T/A (rs1196333) on hepatocellular carcinoma.

In the initial study of Knappskog *et al.*, SNP 285C (rs117039649) was described as an exclusively Caucasian variant when comparing a cohort of Chinese individuals to a Caucasian multi-cohort (Norway, Netherlands, UK and Finland). No data from African or American natives was available though. This variant is in complete linkage with the 309G variant [24]. Here, we report the presence of the 285C variant in the Moroccan population embedded within a single CGT haplotype. The haplotype frequencies, 1% in controls and 1.7% in the HCC group are not significantly different. Whereas the 285C variant is present in almost 12% of North Europeans carrying the 309G variant [25], we found a rate of 4.7% among controls and 6.5% among HCC patients recruited in a Moroccan population. It is known that the 309G variant is enhancing the binding of Sp1 transcription factor to its promoter, increasing therefore MDM2 expression and accelerating tumor formation. Functional studies reported that SNP285C reduces significantly promoter binding by Sp1 attenuating the effect of 309G variant. This observation was consistently made on different sets of tumors (ovarian, endometrial and breast cancers but not lung tumors) in the same ethnic group [24,26]. For the SNP344 (rs1196333), no association was shown between variants and ovarian, endometrial, breast or prostate cancers, despite *in silico* data showing that 344A variant seems to modulate the binding of TFAP2A, SPIB and AP1 transcription factors [20]. The SNP344T is always associated with the SNP309T allele; its status may not affect hepatocellular carcinoma risk (*p* > 0.05).

One interesting feature to discuss in this paper is the allelic distribution of 285G/C and 344A/T. The 285C variant was initially thought to be restricted to North European Caucasian populations and considered as such as a relatively recent polymorphism. This allele is, however, also present in the Moroccan population (1%), a situation that could be explained by a genetic admixture from Western Europe (Spanish, Portuguese or French populations) or by the original presence of SNP285 at this rate in North Africa as it is observed for the habitants of Helsinki in North-Eastern Europe (1.6%). The analysis of SNP285 prevalence in Middle-Eastern population may bring an answer this question. On the contrary, The SNP 344A variant is more frequent in Africans (18% in a group of African Americans) than Caucasians and Asians (3%), and might be an ancient polymorphism evolving similarly to 309 T/G polymorphism [20]. In the present study, we found, as expected, a frequency of 344A allele similar to that measured in Caucasians (4.4%).

These findings are in keeping with the previous observations of Pineau *et al.*, and Ezzikouri *et al.* about the molecular context of HCC in Moroccan patients [16,27]. These authors suggested that given the similarity between populations, the genetic component alone could not provide an explanation the differences of HCC incidence observed between Morocco (0.8 case/10^5^ habitants) or Western North Africa (1 case/10^5^ habitants), and the Northern Bank of the Mediterranean (≥10 cases/10^5^ habitants) [28]. Intriguingly, the prevalence of chronic hepatitis, the principal risk factor for HCC worldwide, is higher in Western North Africa than in most if not all European countries, a situation suggesting that underestimated risk factors present in Europe may provide the decisive thrust towards tumor development [29]. Thus, further areas must be explored, *i.e.* epigenetics, transcriptional and proteomic profiling in line with viral characteristic. Any kinds of data must be gathered in a really careful way to understand the mechanisms triggering liver carcinogenesis in the rather specific context of North African populations known to be submitted to a South–north gradient of sub-Saharan genes but sharing as well an extensive genetic background with them Northern Mediterranean neighbors [30,31].

## Competing interests

The authors declare that they have no competing interest. All the authors read through the article and agreed.

## Authors’ contributions

KR carried out the molecular genetic study, the sequence alignment, the data analysis and drafted the manuscript. SE contributed to the concept of study and the collect of samples. AM, MK and PP contributed to the critical revision of the manuscript. SB contributed to the concept of study, the collect of samples and the critical revision of the manuscript. All authors read and approved the final manuscript.
